# Skin Diseases and Syphilis

**Published:** 1896-08

**Authors:** 


### SELECTIONS AND ABSTRACTS.

### SKIN DISEASES AND SYPHILIS.

EDITED BY DR. M. B. HUTCHINS.

### THE VALUE AND LIMITS OF USEFULNESS OF ELECTROLYSIS IN DERMATOLOGY.

An interesting discussion of this subject was had at the 1895 meeting of The American Dermatological Association, as reported in the Transactions.

There was a little of the usual "Doctors disagree" difference of opinion, but the majority of the members, even while expressing themselves in terms of extra-conservatism, had much to say in its favor from their own experience. A summing up of the views expressed shows the balance in favor of electrolysis in many skin anomalies. Other methods were preferred by the speakers for various conditions amenable to electrolysis, but the position of usefulness of electrolysis seems, to the editor, unshaken.

It appears that those who speak and write upon the subject use too loose terms and that the operations as suggested are not described with sufficient regard for scientific accuracy.

No one should attempt the work without the means of regulating and measuring the current. The current in most cases, to avoid scarring, should be as weak as the requirements of the operation permit. The resistance or susceptibility of each patient must be considered. The work must be done properly, the anatomy, pathology and physical characteristics of the condition and locality being well in mind. The current advised or used should never be expressed in cells, but always in milliamperes. A needle insulated almost to its point is the proper one for growths requiring rather deep punctures. Though a rather tedious method, the ultimate cosmetic results in numerous conditions are such as can be obtained with no other method. That its chief use is in cosmetic operations is admitted, which should enhance rather than diminish its value.

### AMERICAN DERMATOLOGICAL ASSOCIATION.

The meeting will be held at the Hot Springs of Virginia, September 8, 9, and 10, 1896. Everything will be done to make the meeting a success and several papers on interesting subjects have been already promised. Dr. White will open a general discussion on the subject, "What Effect do Diet and Alcohol have upon the Causation and Course of the Eczematous Affections and Psoriasis?" Charles W. Allen, No. 126 East Sixtieth Street, New York, Secretary. This is one of the most scientific bodies in America. It is composed of the majority of the leading dermatologists in the United States and Canada, and its papers and discussions are of great interest and value.

---

FOR severe urticaria give doses of twenty-four grains of salicylate of soda every two hours until three doses are taken.—*Ex.*

